# Factors Impacting Risk Perception under Typhoon Disaster in Macao SAR, China

**DOI:** 10.3390/ijerph17207357

**Published:** 2020-10-09

**Authors:** Yajing Shen, Shiyan Lou, Xiujuan Zhao, Kuai Peng Ip, Hui Xu, Jingwen Zhang

**Affiliations:** 1 Institute of Analytical Psychology, City University of Macao, Macao 999078, China; jstzsyj0401@163.com (Y.S.); dahui2008.love@163.com (H.X.); h18092200174@cityu.mo (J.Z.); 2 Faculty of Finance, City University of Macao, Macao 999078, China; 3 Department of Engineering Physics, Tsinghua University, Beijing 100084, China; 4 Institute of Public Safety Research, Tsinghua University, Beijing 100084, China; 5 Research Center for Macao Social and Economic Development, City University of Macao, Macao 999078, China; kpip@cityu.mo

**Keywords:** typhoon disaster, risk perception, knowledge, information acquisition channels, active response to disaster

## Abstract

Studying typhoon risk perception and its influencing factors help reveal potential risk factors from the perspective of the public and provide a basis for decision-making for reducing the risk of typhoon disasters. The purpose of this study is to assess the risk perception and related factors of Macao residents in China. Information was collected from 983 participants using a structured questionnaire with an effective utilization rate of 94.2%. Descriptive statistics, univariate analysis and correlation analysis were used to analyze the data. The results show that, on the one hand, there are significant differences in risk perception on the factors included: (1) age, education and other demographic characteristics; (2) health status, occupation, length of stay, residence area, residence floor, family organization structure and individuals monthly income and other personal or family conditions; (3) channels and quantity of typhoon information acquisition; (4) degree of mastery of relevant risk aversion knowledge. On the other hand, some factors still have a moderate or high level of correlation with risk perception: (1) The older the respondent, the lower the education level, the lower the income, the lower the risk perception of property damage, health impact and life threat. (2) The more children or elderly people in the family, the higher the risk perception of respondents. (3) The more risk knowledge, the lower the risk perception. (4) The more channels for obtaining information, the lower the fear level and the overall impact of risk perception. (5) The stronger the risk perception, the more positive disaster response behaviors would be taken by the public. In addition, the more information acquisition channels and the less risk knowledge respondents have, the greater the risk perception of the overall impact and the fear of the typhoon; the fewer information access channels and less risk knowledge respondents have, the greater the risk perceptions of property damage, health effects and life threats.

## 1. Introduction

Tropical cyclones are the most destructive disasters in coastal areas together with tsunamis, although the former is more frequent. As a highly destructive weather system, tropical cyclones, whose official warning name is “the wind ball” used in Macao and Hongkong, are cyclonic cyclones that occur in tropical or subtropical oceans, which are also often accompanied by strong winds, heavy rain and storm surges. The tropical cyclone warning signals in Hong Kong and Macao are now divided into: alert signal No. 1, strong wind signal; No. 3, gale or storm signal; No. 8, gale or storm enhancement signal, No. 9; and hurricane signal, No. 10. The World Meteorological Organization (WMO) defines tropical cyclones with central continuous wind speeds between 32.7 m/s and 41.4 m/s as typhoons or hurricanes. In recent years, with global warming, typhoon disasters have occurred frequently, which have caused a serious impact on the entire society [1,2]. In accordance with the recommendations of World Meteorological Organization (WMO), tropical cyclones are classified according to maximum sustained wind near the center of the storm. Based on the 10-min average wind speed, typhoon has been divided into six categories in Macao since 2009, namely, tropical depression, tropical storm, severe tropical storm, typhoon, severe typhoon and super typhoon (Super T.).

**Figure 1:**
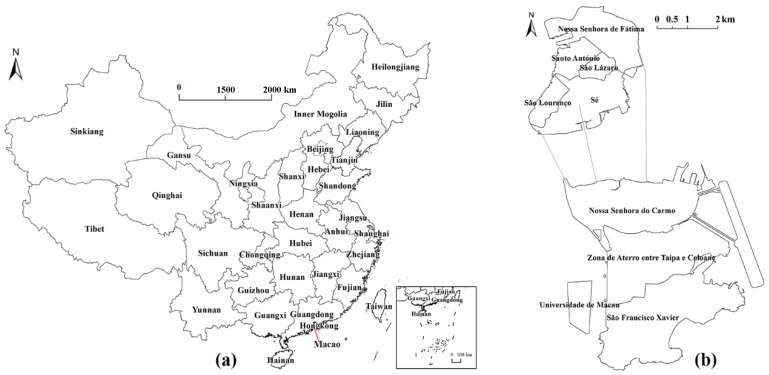
Map. (**a**) Map of China. (**b**) Map of Macao. In 2017 and 2018, the two consecutive years, Macao experienced three super typhoons—Hato, Mangkhut and Yutu—which caused not only economic losses and casualties, but also some psychological trauma to people. Studies found that some people were more vulnerable because they could not respond reasonably or were not good at adapting, while others were the opposite in the face of the same disaster or environmental change [3]. One of the important reasons is that different people have different perceptions of disasters or environmental changes. These differences could perform significant impact on what kind of mitigation decisions will be made by such differences, what mitigation measures will be taken and what will happen to these measures [3,4,5]. Researching people’s perception of disasters is the primary basis for a deep understanding of their adaptation to disasters.

China is one of the countries most severely affected by typhoon disasters. As a coastal area and a low-altitude city in the southeast corner of China, Macao used to have nearly 10 bays, making it one of the most severely affected areas in China (See Figure 1 below for details).

Slovic believes that when applying risk assessment to evacuate hazards, most people rely on intuitive risk judgment, that is, risk perception [6]. He also summarized risk characteristics into unknown risks and fear risks through factor analysis. Sjöberg believes that risk perception refers to a subjective assessment of the probability of a particular accident and how much we are associated with this adverse outcome [7]. He also believes that cognitive risks include both an assessment of probability and the severity of negative outcomes.

Risk perception as a cross-disciplinary and multidisciplinary subject has achieved certain results through decades of research by scholars all over the world. Previous studies have found that public risk perception is related to personal basic conditions, risk knowledge, mass media methods and disaster experience. For example, Lai et al., in a study of Hong Kong, found that women, older respondents and less educated respondents perceived greater environmental risks [8]. Furthermore, researchers found that income has a significant positive effect on risk perception, that is, the higher the income is, the smaller the risk perception will be [9]. Jackson studied earthquake disasters on the west coast of North America and found that the public’s ability to understand disasters is closely related to disaster experience and knowledge of disaster events [10]. Bettman and Park showed that the more comprehensive an individual’s knowledge of risk is, the more it can reduce the uncertainty of risk and the lower its risk perception [11]. Walter et al. found in researching typhoon disasters that “the more people experience typhoon disasters, the higher their perception of risk” [12]. Wei et al. proposed that the number of media reports affect the public’s risk perception based on the theory of public memory [13].

In the field of risk management, some scholars found that there is a significant positive correlation between risk perception and active disaster response behavior [14,15,16]. When studying the coping behavior of the public during typhoons, Riad, Norris and Ruback found that the public’s decision not to evacuate the danger zone is affected by the psychological processes of risk perception and social influence [14]. Matyas, Srinivasa and Cahyanto researched the risk perception and evacuation decisions of tourists in hurricane-affected areas, and found that the higher the risk perception is, the stronger their willingness to evacuate is, and coastal tourists have a higher willingness to evacuate than the mainland tourists [15]. However, some scholars have found that high risk perception does not necessarily result in defensive behaviors in response to natural disasters [17]. Therefore, understanding the public’s perception of disaster risk help us understand why and how they use their own methods to deal with disasters [18].

From the current research status, although in-depth research on risk perception has provided scholars with a wealth of policy recommendations for risk management, the authors found that there is relatively little literature on the risk perception research of typhoon-type meteorological disasters. The predictability makes the public controllable to a certain extent in response to typhoon disasters, which is different from other sudden natural disasters. Under the circumstances of the public having sufficient time to prepare for the risk, their perception of risk and behavioral tendencies need further research.

Therefore, the purpose of this study is to reveal the risk perception and its influencing factors of typhoon disasters among residents of typhoon-stricken areas (Macao) through quantitative research, and to further explore the relationship between public risk perception and enthusiasm for disaster response. Specifically, it analyzed the risk perception and related factors of Macao residents in China, such as demographic characteristics, personal or family conditions, risk knowledge and disaster response behaviors, by testing the following assumptions.

**Hypothesis** **1** **(H1).** *Participants with different demographic characteristics are of significant differences in risk perception.*

**Hypothesis** **2** **(H2).** *Participants in different personal situations are of significant differences in risk perception.*

**Hypothesis** **3** **(H3).** *Participants with different levels of risk knowledge are of significant differences in risk perception.*

**Hypothesis** **4** **(H4).** *The number of typhoon information acquisition channels significantly positively affects participants’ risk perception.*

**Hypothesis** **5** **(H5).** *Participants’ risk perception significantly positively affects their motivation to respond to disasters.*

## 2. Materials and Methods

### 2.1. Study Setting

Macao covers an area of 32.9 square kilometers. By the end of 2019, the population was 679,600, with a population density of 20,657 per square kilometer. “Parish” is its administrative division unit. There are currently seven parishes and one non-parish division area, including Our Lady Fatima Parish, St. Anthony Parish, Sé Freguesias, St. Lazarus Parish, St. Lawrence Parish, Ilha da Taipa, Concelho das Ilhas and Cotai. The lowest point in the region is the sea level of the South China Sea, with an altitude of 0 m. The highest point is Koloane Alto, 172.4 m above sea level. According to the Macao Private Housing Activity Center, the three parishes of Our Lady Fatima Parish, St. Lawrence Parish and Concelho das Ilhas are vulnerable areas to typhoons.

**Figure 2:**
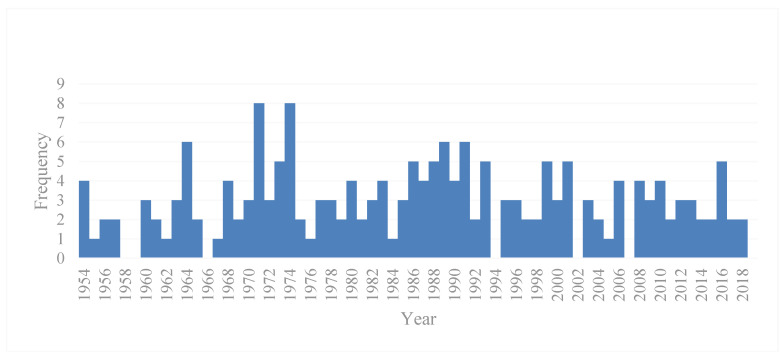
Number of typhoon landings (1954–2019).

Macao has a subtropical monsoon climate, but also has the characteristics of a tropical climate. Spring and summer are humid and rainy, and autumn and winter have low relative humidity and less rainfall. The typhoon season is from May to October, with the most frequent period from July to September. From the number of typhoon landings in Macao from 1954 to 2019, 190 typhoons landed in Macao (only typhoons, severe typhoons, and super typhoons with a maximum average wind of more than 118 km per hour) in 65 years, with an average of 2.92 landings each year. Tropical depressions, tropical storms and severe tropical storms are not included here. Moreover during 2010 to 2019, there were 18 severe typhoons and super typhoons, with an average of twice a year. See Figure 2 and Table 1 for more details.

On 16 September 2018, the strongest typhoon “Mangkhut” wreaked havoc in Macao for more than 24 h, with typhoon No. 10 taking effect for up to 9 h, bringing storms and floods to Macao, especially the low-lying areas, such as the inner harbor and the ocean. Shops were flooded, the infrastructure were damaged and trees fell heavily. “Mangkhut” brought about an estimated direct economic loss of 520 million MOP (65 million USD) and an indirect loss up to 1.03 billion MOP (130 million USD), i.e., a total loss of 1.55 billion MOP (200 million USD). The Macao Civil Defense Center received a total of 445 accident reports and at least 18 people were injured, and one year before, Macao was hit by the strongest typhoon “Hato” since the typhoon observations were made in 1953. (1) Hato was a little bit weaker than Mangkhut, but caused a much more serious damage; (2) Electricity and communications were paralyzed in a large area, and property damage reached 11.47 billion MOP (1.44 billion USD); (3) Most important of all, 10 people were killed and 244 were injured, which was very serious for such a small city as Macao.

### 2.2. Study Participants

The study population needed to have lived in Macao for at least six months, have typhoon experience, either male or female, and have clear consciousness, no mental disorders and the ability to express their views properly. After the investigators’ explanation, they can understand the purpose of our survey, read each question and understand the meaning of their corresponding options and they can make their own choices; or if they cannot read, they can ask investigators to fill it out for them with their own choices after the investigators’ explanations.

A total of 1100 questionnaires were distributed in this study, and 1043 were recovered with a recovery rate of 94.8%. Among them, a total of 983 participants had experienced the typhoons of “Mangkhut” or “Hato”, which is an effective rate of 94.2%.

### 2.3. Data Collection

#### 2.3.1. Survey Design

A structured questionnaire was compiled after consulting relevant literature and conducting preliminary interviews with typhoon experts and local residents. The questionnaire was designed from the following aspects:

(1) Screening items, whether they have experienced typhoon “Mangkhut” or “Hato”;

(2) Demographic characteristics, such as gender, age, education, type of identity, occupation, etc.;

(3) Other personal or family conditions, such as physical health, living area, living floor, length of stay, family structure, personal monthly income, monthly family income, etc.;

(4) Channels to obtain typhoon information, including TV, SMS (Short Message Service), Internet, radio, posting information, newspapers, friends and relatives, loud speakers, etc.;

(5) Risk knowledge, such as understanding of signal lights, knowledge on typhoon and how to prevent possible risks caused by typhoon, and understanding of disaster response behaviors;

(6) Risk perception, examining the participants’ five perceptions of risk before the typhoon—the overall impact, property damage, health effect, life threat, and fear level;

(7) What disaster response actions have been taken?

The five-point Likert scale was used for the two categories of degree judgment and attitude expression. For example, in terms of residents’ awareness of the impact of typhoon disasters, the five items of “overall impact,” “property damage”, “life threat”, “health effect” and “degree of fear”, etc. “Very large/Very strong”, “relatively large/relatively strong”, “average”, “relatively small/relatively weak” to “very small/very weak”, in order of “5, 4, 3, 2, 1”.

#### 2.3.2. Pre-Investigation

In May 2019, members of the research team randomly selected 56 Macao residents who had experienced typhoon disaster to conduct a preliminary survey. The combined reliability of the risk perception part of the questionnaire was 0.9573, and the average variance extracted (AVE) was 0.7907, which had good reliability and validity. Finally, the questionnaire was further modified and improved according to the feedback from the pre-survey. After review by relevant experts, it was revised again to form the final questionnaire.

### 2.4. Statistical Analysis

Statistical analysis was performed using Statistical Product and Service Solutions (SPSS, IBM, New York, NY, USA).

(1) Descriptive statistics was used to describe participants’ demographic characteristics, other personal or family conditions, channels for obtaining typhoon information, risk knowledge, and disaster response behaviors.

(2) Means and standard deviations were used to describe participants’ risk perception scores.

(3) T-test or F-test were used to test whether there are significant differences in risk perception among participants with different demographic characteristics, different personal or family conditions, different levels of knowledge of risk knowledge, and different numbers of typhoon information acquisition channels.

(4) Pearson correlation analysis was used to test whether the five items of risk perception were significantly correlated. Pearson correlation analysis was used to detect whether typhoon knowledge affected risk perception and whether the number of typhoon information acquisition channels affected risk perception.

(5) Variance analysis was used to test whether participants with different risk perceptions had significantly different enthusiasm for disaster response.

## 3. Results and Discussion

In this section, the results are presented and discussed in relation to (1) the distribution of participants’ demographic characteristics and other personal and family conditions; (2) the typhoon information section, which contains the description statistics of the participants’ typhoon disaster prevention knowledge and typhoon information acquisition channels; (3) descriptive statistics of respondents’ risk perception before the typhoon; (4) descriptive statistics of respondents’ active response to typhoon; and (5) multivariable analysis among factors influencing residents’ risk perception.

### 3.1. Demographic Characteristics and Other Information of Participants

#### 3.1.1. Demographic Characteristics of Participants

**Figure 3:**
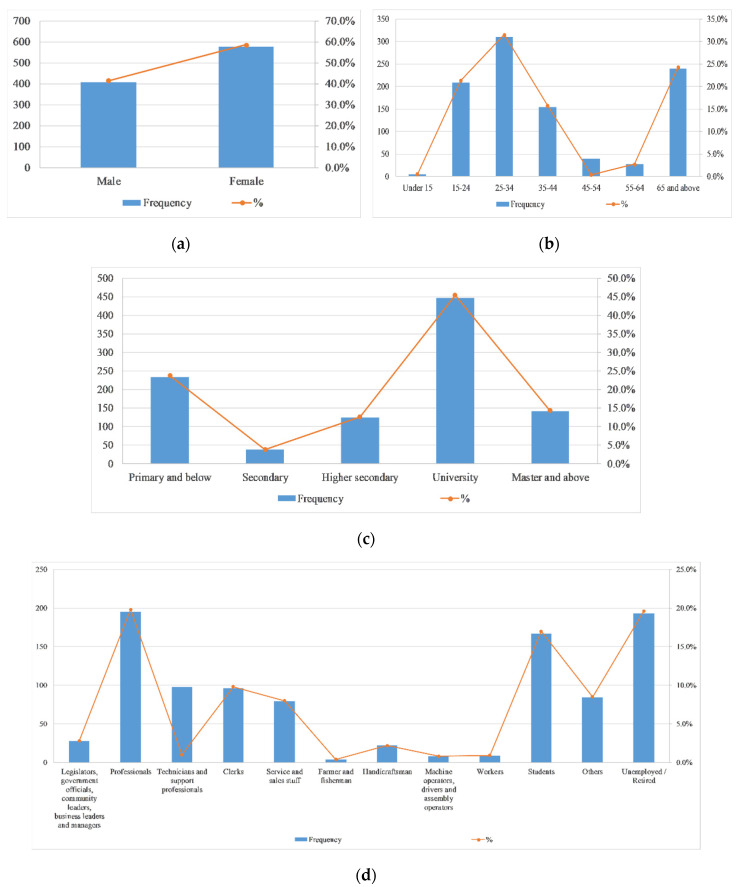
Demographic characteristics of the participants (*n* = 983). (**a**) Gender distribution of participants. (**b**) Age distribution of participants. (**c**) Distribution of participants’ education levels. (**d**) distribution of participants’ occupation.

Figure 3 shows the demographic characteristics of participants. The proportions of men and women in the participants were basically the same, with more people in the 25–30 and 65-year-old groups. Nearly half of the participants with a college degree in the valid questionnaire recovered. In terms of occupational distribution, there are more professionals (19.8%), students (17%) and unemployed persons (19.6%) than other occupations.

#### 3.1.2. Other Personal Information of Participants

**Figure 4:**
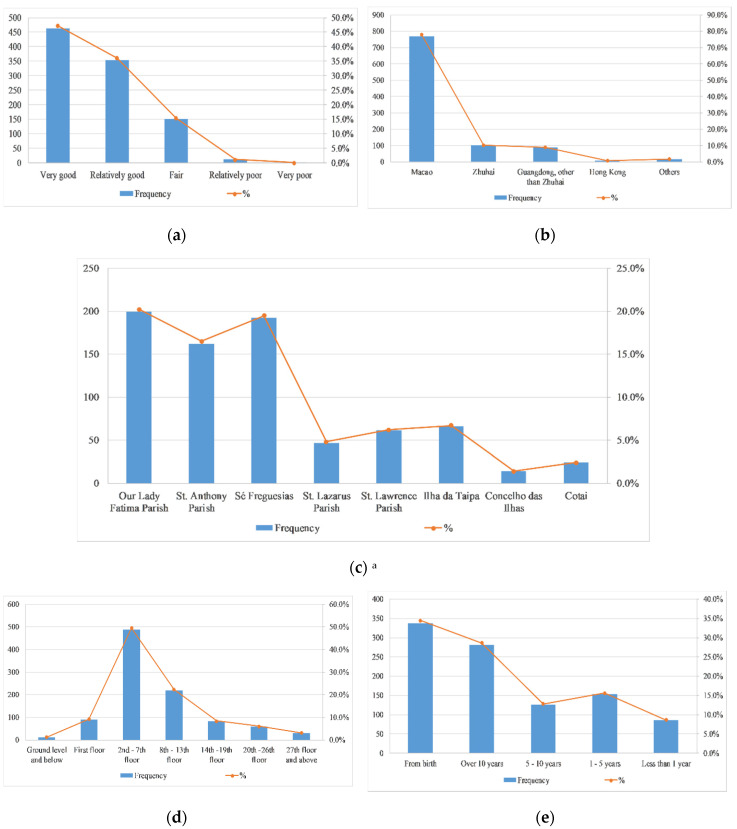

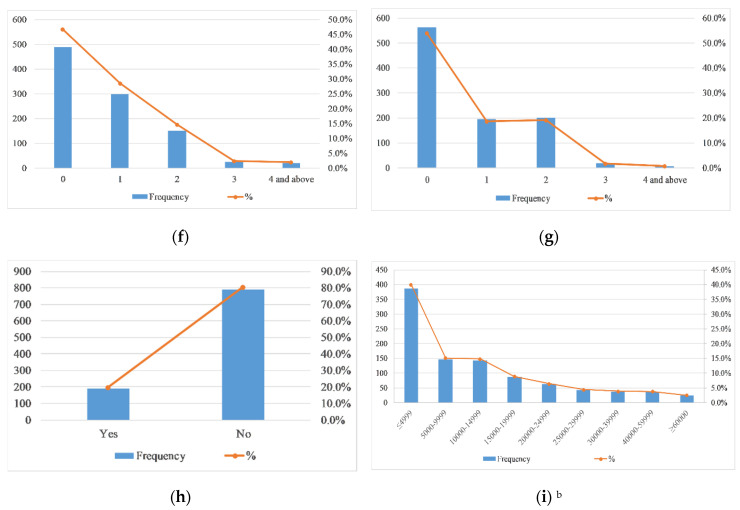
Other personal information of participants (*n* = 983). (**a**) Distribution of participants’ health condition. (**b**) Distribution of participants’ place of residence. (**c**) Distribution of participants’ residential zone. (**d**) Distribution of participants’ living floors. (**e**) Distribution of participants’ length of stay. (**f**) Distribution of the number of family members under the age of 14 of the participants. (**g**) distribution of participants. (**h**) distribution of participants. (**i**) distribution of participants. ^a^ If the place of residence is outside Macao, skip this question; ^b^ missing system 16.

Figure 4 shows that more than 80% of the participants consider their health condition relatively good (36.1%) or very good (47.2%). There are a total of eight parishes in Macao: Our Lady Fatima Parish, St. Anthony Parish, Sé Freguesias, St. Lazarus Parish, St. Lawrence Parish, Ilha da Taipa, Concelho das Ilhas and Cotai. A total of 78.2% of the participants lived in Macao and most of the participants in Macao lived in the following three parishes: Our Lady Fatima Parish (20.2%), St. Anthony Parish (16.5%) and Sé Freguesias (19.5%). When it comes to living floors, nearly half of the participants said they lived on the second to seventh floors. Due to our initial consideration of the structure of the questionnaire, most of the respondents stated that they were native Macao people (34.4%) or had been living in Macao for more than ten years (28.6%). When it comes to family structure, the majority of respondents said there was no family member with the age of under 15 years of age (46.7%) or over 65 years old (54.0%), and 80.4% of respondents said they had no family members with mobility problems. In terms of economic status, 40% of the respondents had a personal monthly income below 5000 MOP (~600 USD), 29.9% of the respondents had a personal monthly income between 5000 MOP (~600 USD) and 15,000 MOP (~1800 USD).

### 3.2. Typhoon Information

#### 3.2.1. Knowledge of Typhoon Disaster Prevention

**Figure 5:**
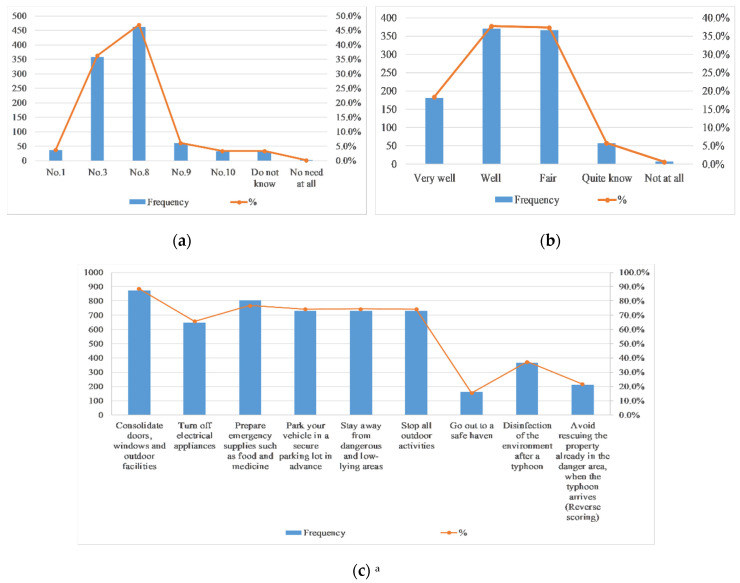
Participants’ knowledge of typhoon disaster precautions (*n* = 983). (**a**) Distribution of “When the typhoon signal is _ or above, one should get prepared for typhoon in advance.” (**b**) Distribution of “Do you know how to guard against typhoons?” (**c**) Distribution of “Which of the following measures do you think will reduce your loss?” ^a^ Percentage total may add up to more than 100% as multiple responses were permissible.

Many scholars have shown in their research that improving residents’ knowledge of risk aversion helps to reduce the potential risks of typhoons [19]. It is officially stipulated that the suspension of typhoon signal (i.e., wind ball) No. 8 and above requires advance prevention and paid holidays. As is shown in Figure 5, only 47% of the respondents believe that a typhoon with a typhoon signal No. 8 and above need to be prepared in advance. Although more than half of the respondents indicated that they knew well (37%) or they were very familiar with (18.4%) precautions, 21.8% of the respondents stated that “when typhoon comes, rescuing property already in dangerous areas” can reduce losses, which is actually a risky activity. Most respondents believe that measures to reduce losses are pre-disaster preparations, such as consolidating doors, windows and outdoor facilities (88.6%), turning off electrical appliances (65.8%), preparing emergency supplies, such as food and medicine (76.8%), parking their own vehicle in a secure parking lot in advance (74.2%) and staying away from dangerous and low-lying areas (74.4%).

#### 3.2.2. Information Channels on Risk Communication

**Figure 6:**
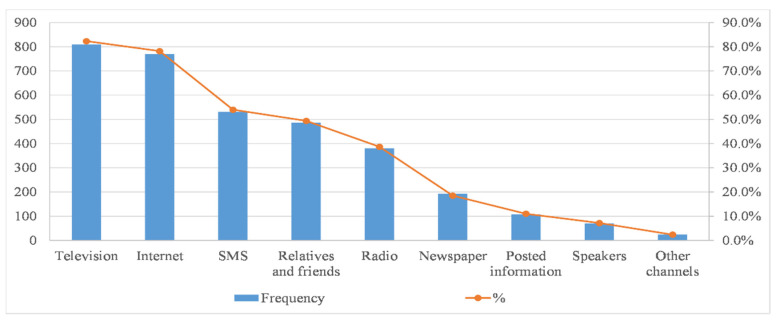
Participants’ information channels for typhoon (*n* = 983) ^a^. ^a^ Percentage total may add up to more than 100% as multiple responses were permissible.

Figure 6 shows that TV (82.3%) and the internet (78.2%) were the main channels for most residents to obtain typhoon information before the typhoon arrives. Furthermore, the respondents also received the information through SMS (54.0%), radio (38.7%) or friends or relatives (49.4%). This result basically coincides with AlQahtany’s results, which revealed that television, mobile phone text message and the internet rank among the top three existing methods of obtaining information about disaster risks [20]. Compared with other research, in addition to traditional media–television, the internet has become a very important channel for people to obtain typhoon information [13,21,22,23,24,25].

### 3.3. Risk Perception of Residents before Typhoon

**Figure 7:**
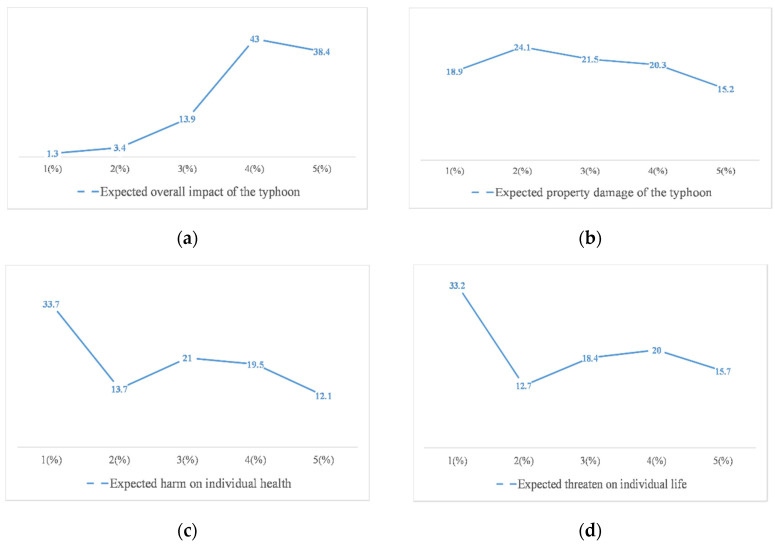

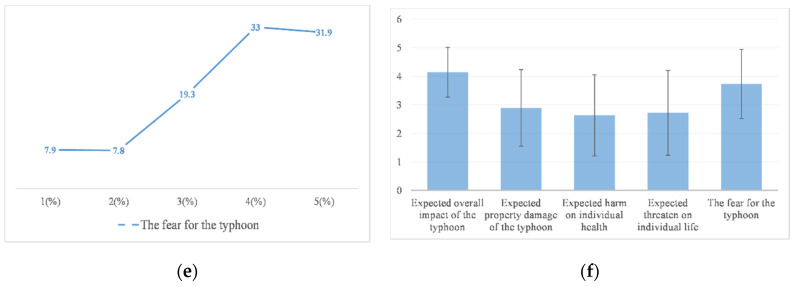
Knowledge, information channels and active response to typhoon and risk perception. (**a**) Distribution of participants’ expected overall impact of typhoon. (**b**) Distribution of participants’ expected property damage of typhoon. (**c**) Distribution of participants’ expected harm on individual health. (**d**) Distribution of participants’ expected threaten on individual life. (**e**) Distribution of participants’ fear of typhoon. (**f**) The mean (M) and standard deviation (SD) of different dimension of risk perception.

Figure 7 shows respondents’ risk perception of typhoon before its landfall with regard to overall impact, property damage, health effect, life threat and fear level. A total of 81.4% of them thought the overall impact of typhoon was big or very big (scored 4 or 5 out of maximum 5). About one-third of respondents had a high-risk perception of the risks associated with property damage, health effects, and life threats, while, 64.9% of them indicated that their fear level was high or very high. From an average point of view, the overall impact and fear level of respondents on risk perception, which was around 4, was higher than other three dimensions, including property damage, life threatening and health effects, with scores ranging from 2.5 to 3.

### 3.4. Active Response for Typhoon

**Figure 8:**
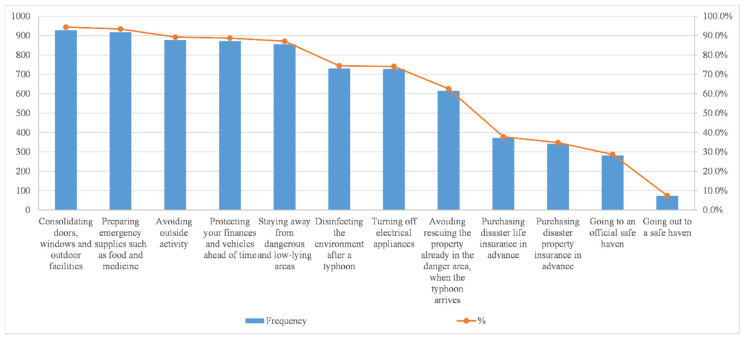
Participant’s active response to typhoon (*n* = 983) ^a^. ^a^ Percentage total may add up to more than 100% as multiple responses were permissible.

As is shown in Figure 8, the top five measures for active disaster response are: consolidation of doors, windows and outdoor facilities (94.4%), preparation for emergency supplies such as food and medicine (93.4%), avoidance of outside activity (89.2%), protection of personal wealth and vehicles ahead of time (88.7%), staying away from dangerous and low-lying areas (87.1%). In the previous study of Typhoon Mangkhut by Chan et al., similar conclusions were reached [26]. However, the number of people who choose to go out to a safe haven is the least, only 7.4%. This may be due to the government’s stipulation that if the hoisting of No. 8 typhoon signal is announced during work hours, workers should leave work in batches, and the government will give a notice two hours in advance to facilitate smooth traffic. If it is suspended before work in the morning, companies and schools will automatically suspend work and classes, and the Hong Kong stock market will also be suspended. When Typhoon No. 8 turns into a weaker Typhoon No. 3, employees need to return to the company within two hours.

### 3.5. Factors Influencing Residents’ Risk Perception—Multivariable Analysis

Pearson correlation analysis was used to test five topics related to risk perception, and Table 2 below shows that a significant positive correlation between each of them at *p* < 0.001. Among them, the degree of fear and property damage have a higher impact on the risk perception of the overall impact of the respondents, with correlation coefficients of 0.369 and 0.330, respectively. Moreover, there is a strongly significantly positive correlation between the respondents’ perceptions of the risk of property damage and their risks to health and life threats, with correlation coefficients of 0.777 and 0.701, respectively. The correlation coefficient for health effect and life threat risk perception is also as high as 0.822. First of all, health and life are closely related to each other. It is generally believed that threats to life generally endanger health, so the two have a strong positive correlation. Furthermore, once the typhoon has an impact on health or life, the individual’s property will naturally suffer from a corresponding loss.

#### 3.5.1. Comparison Analysis of Risk Perception based on Demographic Characteristics and Correlation Analysis between Them

(1) Risk perception about overall impact

As is shown in Table 3, respondents of different ages had significant differences in their perception of the overall impact of the typhoon before typhoon arrives at *p* < 0.05, while respondents of different occupations had significant differences in their perception of the overall impact of the typhoon before the onset of the typhoon at *p* < 0.01. Among them, middle-aged respondents (35–54 years old) had a higher average risk perception of the overall impact of typhoons, which was about 4.3. When the individual is 35 to 59 years old, that is, after entering middle age, the physical and behavioral maladjustment and psychological imbalance will occur, which is what we often call the “middle age crisis”. Middle-aged people are generally the backbone of the family and have heavier family responsibilities, because they need to not only raise their children, but also support their parents. This may cause respondents in this age group to pay more attention to the overall impact of typhoons, so their average risk perception score is higher. As far as occupations are concerned, only the risk perception of the overall impact of typhoons of service and sales and students are less than four points, which may be because such respondents who are mainly immigrant labors or students from Mainland China have not stayed at Macao for a long time. They do not have a sense of regional belonging, so they were not very concerned about the impact of the typhoon. Or, maybe because most of them are from inland and have not experienced typhoon before.

(2) Risk perception about property damage

As is shown in Table 3, respondents of different genders, ages, education and occupations have significant differences in their perception of risk to property damage, at *p* < 0.001. In terms of gender, men’s risk perception scores for property damage are significantly higher than women’s, which may be because men bear more household expenses; this result is contrary to Lai’s research on Hong Kong [8]. The 25–44 age group perceived a greater risk of property damage than other age groups, with the age group over 65 years the least aware of it. From the perspective of education, respondents with a higher secondary education have a higher risk perception of property damage and the unemployed have the lowest risk perception score of property damage, which may be because they do not typically own as much property. This is basically consistent with previous research results [27].

(3) Risk perception about health effects

As is shown in Table 3, respondents of different genders, ages, education and occupations have significant differences in their perception of risk to health effects, at *p* < 0.001. The differences between the subgroups are basically consistent with the dimension of property damage, and will not be repeated here.

(4) Risk perception about life threat

As is shown in Table 3, respondents of different genders, ages, education and occupations have significant differences in their perception of risk to life threat, at *p* < 0.001. The differences between the subgroups are basically consistent with the dimensions of property damage and health effect, and will not be repeated here.

(5) Risk perception about fear level

As is shown in Table 3, respondents of different genders, ages, education and occupations have significant differences in their perception of risk to fear level, at *p* < 0.001. Different from the results in the previous dimensions, respondents over the age of 65, those with primary education and below have a significantly higher level of fear of typhoon risk than other groups. This may be due to the low level of education of this group, the lack of knowledge of risks, the inexplicable fear of natural disasters and the uncertainty in how to reduce the risks and the extent of losses.

In summary, Hypothesis 1, that “participants with different demographic characteristics are of significant differences in risk perception” is accepted.

(6) Correlation between demographic characteristics and risk perception

As is shown in Table 3, age and education are related to risk perception. Regarding age, on the one hand, there is a significant and moderate negative correlation between age and the risk perception of property loss, health impact and life threat with the correlation coefficients of −0.343, –0.442 and −0.469, respectively. On the other hand, there is a significant low-degree positive correlation between age and the degree of fear, the risk perception, with a correlation coefficient of 0.147. In other words, to a certain extent, the older the respondent, the lower the risk perception of the respondents in terms of property damage, health impact and life threats. This conclusion is contrary to the findings of Lai et al. on Hong Kong, which may be due to Macao’s unique social welfare system [8]. Macau’s social welfare system can guarantee the livelihoods of its residents: for instance, in 2018, each elderly person can receive 69,850 MOP (8728 USD) as pension or cash sharing from the government, which is enough for their daily use. Furthermore, the Macao government pays much attention to the residents’ livelihood. Therefore, the elderly residents know that, even if the typhoon has an impact on their lives, the government will provide subsidies in time, so their risk perception is relatively low.

Regarding education, on the one hand, there is a significant and moderately positive correlation between education and the risk perception of property loss, health impact and life threat, and the correlation coefficients are 0.325, 0.413 and 0.421, respectively. On the other hand, there is a significant low-degree negative correlation between education and the degree of fear in terms of risk perception, with a correlation coefficient of −0.165. Most of the locals or the elderly in Macao have relatively low academic qualifications, so this result can also be partly attributed to Macao’s unique social welfare system.

#### 3.5.2. Comparison Analysis of Risk Perception based on other Personal or Family Circumstances and Correlation Analysis between Them

(1) Risk perception about overall impact

As can be seen from Table 4 that respondents with different health conditions have significantly different risk perceptions of the overall impact of the typhoon, at 1% level. Respondents living in hazardous areas have a significantly higher level of risk perception of the overall impact. The presence of elderly people over 65 or family members with limited mobility at home also affect respondents’ perception of the risk of overall impact, at *p* < 0.05. Respondents who live with family members with limited mobility have a higher level of risk perception of the overall impact of the typhoon. This is also very easy to understand. After all, if there are elderly people or members with limited mobility in the family, their evacuation behavior in case of disaster will definitely be affected.

(2) Risk perception about property damage, health effects and life threat

Due to the high correlation between these three dimensions of property damage, health effects and life threats, there is also some consistency in the analysis of them and other variables, and they are analyzed together to prevent redundancy.

All the personal and family conditions, including health condition, place of residence, whether to live in areas susceptible to typhoon, living floor, length of stay, monthly personal income, monthly family income and whether there are children under 14 years old, elderly over 65 years old or family members with limited mobility affected the respondents’ risk perception of property damage, health effects and life threat, at 5%, 1% or 0.1% level.

First of all, respondents with poor health feel greater threats to property, health and life, which may be due to the fact that these the respondents believe that once they are in danger, their lives will be worse. Secondly, residents living in Macao have the lowest level of risk perception in these three dimensions. Previous studies have also shown that in areas like Macao where typhoon occur more frequently (almost every year there are several heavy rains and typhoons), residents’ risk perception is low, since although the previous incidents also caused some losses, the fatality rate was low and personal property losses were low, which caused a sense of control among residents to a certain extent [19,28]. On the one hand, it may be because local residents have become accustomed to typhoons; on the other hand, typhoons have never directly hit Macao in preceding years, which made them think that Macao is a blessed place. Third, respondents who live in areas susceptible to typhoons or live on the ground floor and below are more aware of the risks of property damage, health and life threats, which is the same as Alqahtany’s study [20]. This is obvious because these areas or floors are more likely to be flooded. Fourth, respondents who stayed in Macao for five to ten years are more aware of these three risks, which may involve Macao policies. The Macao government requires a time span of 7 years for both skilled and married immigrants who have just obtained their non-permanent residents identity to become permanent residents and enjoy the benefits for permanent residents. Therefore, some of the respondents who have stayed in Macao for five to ten years are about to acquire or have just obtained permanent resident status on the basis that they have accumulated a certain amount of wealth, so the level of perception of these three risks is higher. Fifth, people with the weakest perception of these three risks include respondents who do not have children under 14 years of age, elderly people over 65 years of age or members with limited mobility. After all, such a family structure needs to be considered when taking risk avoidance measures or making decisions. Finally, when it comes to personal income, respondents with an income of less than 5000 MOP (600 USD) have the weakest perception of these three risks, probably because they have nothing to lose, or even if they want to prevent losses, there is no financial support. This result is the same as Kellens, who thought that these people may not consider the risk problem because of the risk avoidance cost [29]. Respondents with incomes ranging from 10,000 MOP (1254 USD) to 14,999 MOP (1881 USD) have the strongest risk perception in these three categories, probably because this group of people has just started their careers and does not want to lose anything due to natural disasters, which will affect their future development.

(3) Risk perception about fear level

Living floor, length of stay, monthly family income and whether there are family members with limited mobility are all factors impacting the respondents’ risk perception of life threat, at 5%, 1% or 0.1% level. First of all, respondents living in areas prone to typhoons and on the ground floor and below have a higher degree of fear, mainly of heavy rains and floods that follow the typhoon to overwhelm houses or roads. Second, respondents who had stayed in Macao for less than one year had the highest level of fear, probably because they were not familiar with typhoons. Finally, the interviewees with family members with limited mobility have the highest degree of fear. The reasons have been stated above and will not be repeated here.

In general, hypothesis two that “participants in different personal situations are of significant differences in risk perception” is accepted.

(4) Correlation between personal monthly income and risk perception

Further analysis on Spearman’s correlation of other personal information and various dimensions of risk perception found that, first of all, the length of stay is significantly negatively correlated with the risk perception of the three dimensions of property damage, health effect and life threatening, and the correlation coefficients for these are −0.126, −0.213, and −0.208, respectively. In other words, the longer a resident stays in Macao, the lower the risk perception. However, the length of stay is positively correlated with the degree of fear. The shorter the respondent stays in Macao, the more fearful they feel.

Second, number of family members under 14 or above 65 is significantly positively correlated with risk perception in the three dimensions of property damage, health effect and life threat, with correlation coefficients greater than 0.35, which also means that the more family members under 14 or above 65, the greater the risk perception. Moreover, there is a significant but lower positive correlation between the number of family members over 65 years of age and the risk perception of the overall impact.

Third, research shows that the higher the income, the stronger the risk perception of property damage, health effects and life threats. Although this result is contrary to Spence, we believe that respondents with higher incomes cherish their property, health and life more and therefore have a higher level of risk perception [9].

#### 3.5.3. Comparison Analysis of Risk perception based on Knowledge and Correlation Analysis between Them

(1) Knowledge of typhoon signal

Respondents with different degrees of typhoon signal understanding have significant differences in the two risk perception dimensions of overall impact and degree of fear, at 5% or 0.1% level, while the understanding has no significant correlation with risk perception. There are no significant differences in the three risk-related dimensions of property damage, health effect and life threatening.

(2) Understanding of typhoon prevention

The respondents’ understanding on preventive measures is significantly different in all five dimensions of risk perception. Not only that, but there is a significant negative correlation in the risk perception of the three dimensions of property damage, health effect and life threat; the correlation coefficients are −0.182, −0.138 and −0.120, respectively. In other words, the more they know about the preventive measures for typhoon, the lower their risk perception. This conclusion is consistent with Bettman, since the more comprehensive knowledge that an individual has, the lower the uncertainty of the risk and the lower the risk perception [11].

(3) Knowledge of typhoon preparedness

This item is basically consistent with the results of the previous project which shows that respondents with different knowledge of typhoon preparedness have significant differences in risk perception in five dimensions, at 1% or 0.1% level. In addition to the overall impact and the degree of fear, the risk perception of the other three dimensions is significantly negatively correlated with knowledge of typhoon preparedness; the correlation coefficients are −0.187, −0.202 and −0.213, respectively, which is slightly higher than understanding of typhoon prevention. Obviously, the deeper you understand the preventive measures, the lower your perception of risk. After all, if residents know the preventive measures for typhoons well, they know how to avoid risks from the aspects of property, health and life, and minimize the risk of typhoons. The corresponding risk perception will naturally decrease. Moreover, if residents do not know how to prevent typhoons, they may not be able to accurately estimate the risk of loss, and may easily overestimate the loss.

In summary, hypothesis three that “participants with different levels of risk knowledge are of significant differences in risk perception” is accepted.

#### 3.5.4. Comparison Analysis of Risk Perception based on Information Channels and Correlation Analysis between Them

As can be seen from Table 5, the number of information acquisition channels has a statistically positive impact on the respondents’ risk perception, at 1% or 0.1% level. Moreover, the more information the interviewees have access to before the typhoon, the lower their level of risk perception of health effects and life threat. These findings are consistent with the research of Bettman and Park [11]. Meanwhile, these are exactly the opposite in terms of overall impact and fear. There is a positive correlation between the level of fear and the number of channels for obtaining information; the correlation coefficient is 0.246, at 0.1% level. This may be due to the amplification mechanism of risk. Slovic proposed that the magnitude of the impact of a risk event depends not only on the nature of the risk event, but also on how the public obtains relevant information and how to perceive and explain these messages during the risk communication [6]. Furthermore, based on public memory theory, Wei et al. suggested that the number of media reports affect the public’s perception of risk [13].

In summary, hypothesis four that “the number of typhoon information acquisition channels significantly positively affects participants’ risk perception” is accepted.

#### 3.5.5. Comparison Analysis of Active Response to Typhoon based on Risk Perception and Correlation Analysis between them

As can be seen from Table 5, risk perception in different dimensions has statistically significant impact on active disaster response behavior, at *p* < 0.001. Further correlation analysis showed that the greater the perceived risk, the more proactive measures the residents would take, which matches the previous study. Matyas, Srinivasa and Cahyanto conducted a study on risk perception and evacuation decisions of tourists in hurricane-affected areas, and found that the higher the risk perception, the stronger the evacuation willingness [15]. In addition, in the field of risk management, some scholars found that there is a significant positive correlation between risk perception and positive disaster response behavior [14,15].

In summary, hypothesis five that “participants’ risk perception significantly positively affects their motivation to respond to disasters” is accepted.

#### 3.5.6. Coupling Analysis of Knowledge, Information channels and Risk perception

**Figure 9:**
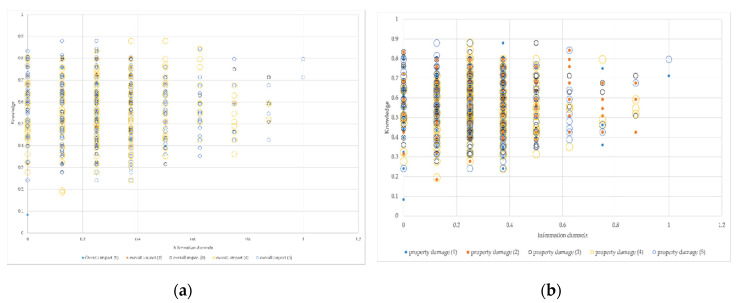

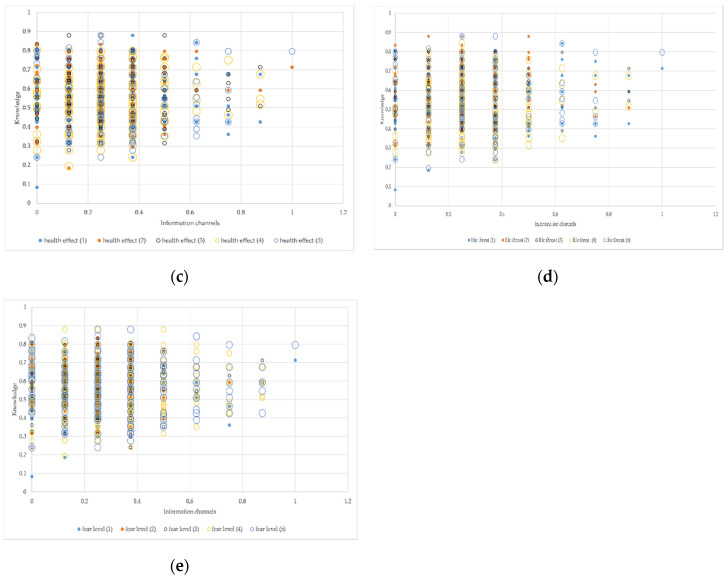
Coupling analysis of knowledge, information channels and risk perception. (**a**) Coupling analysis of knowledge, information channels and risk perception of the overall impact. (**b**) Coupling analysis of knowledge, information channels and risk perception of the property damage. (**c**) Coupling analysis of knowledge, information channels and risk perception of the health effect. (**d**) Coupling analysis of knowledge, information channels and risk perception of the life threat. (**e**) Coupling analysis of knowledge, information channels and risk perception of the fear level.

As can be seen from the following scatter diagram Figure 9, first of all, the more information acquisition channels and the less risk knowledge respondents have, the greater the risk perception of the overall impact of the typhoon; secondly, the fewer information access channels and less risk knowledge respondents have, the greater risk perceptions of property damage, health effects and life threats; finally, the more information access channels and the less risk knowledge respondents have, the higher their fear of typhoons.

## 4. Conclusions and Prospects

### 4.1. Conclusions

Although this research has certain shortcomings, it has some theoretical and practical significance in the field of risk perception. First of all, with questionnaires and statistical methods, such as hypothesis testing and correlation analyses, this paper studied such factors as demographic characteristics, personal or family circumstances, hedge knowledge, access to information and so on, are taken into consideration that may have an influence on public’s risk perception.

(1) Judging from the average of the five dimensions of risk perception, the highest degree of public perception is the overall impact of typhoon risk (M = 4.14), followed by the degree of fear (M = 3.73) and then the property damage (M = 2.89), Life threat (M = 2.72) and health effect (M = 2.63).

(2) The more knowledge and preventive measures for typhoon, the lower the risk perception, most of which are reflected in the three dimensions of property damage, health effect and life threat. This result is consistent with the study of Bettman and Park [11].

(3) The more typhoon information is obtained, the stronger the public’s overall risk perception and fear of the typhoon, and the weaker the risk perception of health effects and life threats. This result is consistent with the study of Wei et al. [12].

(4) The stronger the risk perception, the more positive disaster response behaviors the people adopt, which is consistent with the researches of Riad et al. and Matyas et al. [14,15]. Furthermore, the positivity of the people should be mainly reflected in the prevention before the disaster.

(5) There are significant differences in the perception of typhoon risk among people of different age groups. The risk perception of the typhoon of the 25–44 age group is significantly higher than that of the other groups. This result is reflected in the five dimensions of risk perception. The age group of over 65 years of age has a significantly lower risk perception of property damage, life threats and health effects than the other groups. From the perspective of the correlation between age and risk perception, age has a significant negative correlation with the risk perception of property damage, health effects and life threats.

(6) Respondents with different education have significant differences in risk perception of the four dimensions of property damage, life threat, health effect and degree of fear. The risk perception of respondents in the high school education in the three dimensions of property damage, life threats, and health effects is significantly higher than those of the other groups; respondents with primary school education and below have a significantly lower risk perception in these three dimensions than other groups, but their fear levels are significantly higher than other groups. In terms of the correlation between education level and risk perception, there is a significant positive correlation between education level and risk perception of property damage, health impact and life threats.

(7) Respondents of different occupations have significant differences in typhoon risk perception. From the perspective of overall impact, legislators, government officials, community leaders, business leaders and managers, and handicrafts-man have higher risk perceptions, and service and sales staff and students have lower risk perceptions; the risk perception in the three dimensions of property damage, health effect and life threats of the three occupations of professionals, technicians and support professionals, and handicrafts workers is higher and of the unemployed is lower in the degree of fear. People of other occupations are of the highest degree of fear and students are of the lowest degree. In terms of the correlation between health status and risk perception, although there is a significant correlation, the correlation coefficient is low and can be ignored.

(8) Respondents with different personal and family situations also showed significant differences in typhoon risk perception from various dimensions. In terms of living floors, respondents living on the ground floor or below have higher risk perception; in terms of health, respondents with poor physical condition have higher risk perception; in terms of cities of residence, respondents living in Macao have the lowest risk perception, and respondents who live in areas vulnerable to typhoons have a higher risk perception; in terms of length of stay, the longer the residents stay, the higher their risk perception is in the three dimensions of property damage, health effect and life threat, and the lower the degree of fear is; in terms of the structure of family members, respondents with children under 14 years of age, elderly people over 65 years of age or members with reduced mobility have a stronger risk perception; finally, in terms of personal monthly income, the higher the income, the stronger the risk perception of property damage, health effects and life threats.

Secondly, this research is the first to analyze risk perception from five aspects: overall impact, property damage, health impact, life threat, and degree of fear. Furthermore, the results revealed that although there is a high degree of correlation between various aspects of risk perception, when it comes to the relationship between them and other factors (such as personal circumstances, risk knowledge, or typhoon information acquisition channels, etc.), there are still some differences.

Thirdly, the existing research on risk perception, the demographic characteristics involved only includes basic information such as gender and age, while this research involves more further personal conditions such as residential area, residential floor and family member organizational structure. In comparison, the research on the factors affecting risk perception has a wider coverage.

Fourthly, the more information acquisition channels and the less risk knowledge respondents have, the greater the risk perception of the overall impact of the typhoon; the fewer information access channels and less risk knowledge respondents have, the greater the risk perceptions of property damage, health effects and life threats; the more information access channels and the less risk knowledge respondents have, the higher their fear of typhoons.

Finally, although the study uses Macao as the research area, most of the research results are consistent with previous research results. Therefore, the results of this study can be generalized and applied to most coastal cities or island cities.

### 4.2. Prospects

This research only studies the influencing factors of public risk perception of typhoon. Future research can be extended from the following three aspects:

Firstly, fully consider the role of risk information dissemination on public risk perception. With the help of big data platform, we can obtain massive risk information and public preference data of diversified communication media after the occurrence of emergencies, explore the characteristics of public information demand and measure the psychological gap caused by the difference between public demand information and risk information. On this basis, with the help of behavioral decision theory and psychological theory, the public’s risk perception is portrayed, and according to the public’s different preferences of the media, the change law of risk perception can be studied.

Secondly, the life cycle factors of emergencies can be introduced in the description of risk perception. By refining the life cycle of emergencies, scholars can conduct a phased study of public risk perception, predict the public’s psychological expectations based on the different phases of the event situation, and refine the influencing factors and mechanism of public risk perception, then measure public psychological perception at various stages based on this. Further, by considering the influencing factors of the evolution of different stages of the life cycle of emergencies, the evolution of public psychological perception between different stages can be studied.

Thirdly, on the one hand, follow-up research on risk perception can take the system trust into consideration, including expert trust, market trust, government trust and media trust; on the other hand, interest-related factors may also affect individual risk perception. Benefits include not only positive benefits but also negative benefits, and perspective of long-term or short-term benefits can be considered. For example, in the results of this article, it is found that some respondents who have lived in Macao for 5–10 years have stronger risk perception, which may be related to the permanent residence policy of Macao, which may be considered as an interest-related factor.

## Figures and Tables

**Table 1:** Statistics of Severe Typhoons and super typhoons (2010–2020).

Type	Name	Month
Severe Typhoon	Fanabi	September 2010
Super Typhoon	Megi	October 2010
Severe Typhoon	Nigg	October 2011
Severe Typhoon	Vicente	July 2012
Severe Typhoon	Tembin	August 2012
Super Typhoon	Utor	August 2013
Super Typhoon	Usagi	September 2013
Severe Typhoon	Krosa	Nov. 2013
Super Typhoon	Rammasun	July 2014
Severe Typhoon	Mujigea	October 2015
Super Typhoon	Meranti	September 2016
Severe Typhoon	Megi	September 2016
Super Typhoon	Sarika	October 2016
Super Typhoon	Haima	October 2016
Super Typhoon	Hato	August 2017
Severe Typhoon	Khanun	October 2017
Super Typhoon	Mangkhut	September 2018
Super Typhoon	Yutu	October 2018
Severe Typhoon	Wutip	February 2019
Severe Typhoon	Lekima	August 2019
Severe Typhoon	Lingling	September 2019
Severe Typhoon	Hagibis	October 2019
Severe Typhoon	Bualoi	October 2019
Super Typhoon	Halong	Nov. 2019
Severe Typhoon	Kammuri	Dec. 2019

**Table 2:** Correlation between items of risk perception.

		Overall Impact	Property Damage	Health Effects	Life Threat	Fear Level
Overall impact	Pearson	1	-	-	-	-
Property damage	Pearson	0.330 ***	1	-	-	-
Health effects	Pearson	0.251 ***	0.777 ***	1	-	-
Life threat	Pearson	0.207 ***	0.701 ***	0.822 ***	1	-
Fear level	Pearson	0.369 ***	0.298 ***	0.291 ***	0.316 ***	1

*** *p* < 0.001.

**Table 3:** Demographic characteristics & Risk perception.

Item	Option	Overall Impact			Property Damage			Health Effect			Life Threat			Fear Level		
		Mean ± SD	t/F ^a^	r ^b^	Mean ± SD	t/F ^a^	r ^b^	Mean ± SD	t/F ^a^	r ^b^	Mean ± SD	t/F ^a^	r ^b^	Mean ± SD	t/F ^a^	r ^b^
**Gender**	Male		1.869		3.35 ± 1.312	9.592 ***		3.20 ± 1.296	11.200 ***		3.30 ± 1.341	10.866 ***			−1.373	
	Female				2.56 ± 1.261			2.22 ± 1.372			2.31 ± 1.447					
**Age**	15–24	4.00 ± 1.010	2.316 *	0.048	2.86 ± 1.340	41.738 ***	−0.343 **	2.75 ± 1.343	63.902 ***	−0.442 **	2.88 ± 1.414	70.483 ***	−0.469 **	3.35 ± 1.304	7.547 ***	0.147 **
	25–34	4.13 ± 0.875			3.36 ± 1.284			3.23 ± 1.325			3.41 ± 1.318			3.77 ± 1.095		
	35–44	4.29 ± 0.747			3.46 ± 1.274			3.23 ± 1.262			3.28 ± 1.331			3.84 ± 1.099		
	45–54	4.31 ± 0.655			3.18 ± 1.355			2.67 ± 1.243			2.56 ± 1.392			3.31 ± 1.436		
	55–64	3.89 ± 0.892			2.70 ± 1.409			2.48 ± 1.397			2.63 ± 1.275			3.44 ± 1.219		
	65 and above	4.17 ± 0.815			1.90 ± 0.763			1.35 ± 0.790			1.35 ± 0.826			4.04 ± 1.210		
**Education**	Primary and below		1.134	−0.008	1.93 ± 0.789	52.355 ***	0.325 **	1.35 ± 0.833	89.754 ***	0.413 **	1.38 ± 0.896	93.219 ***	0.421 **	4.11 ± 1.159	9.925 ***	−0.165 **
	Secondary				2.92 ± 1.689			2.70 ± 1.579			2.76 ± 1.480			3.38 ± 1.460		
	Higher secondary				3.33 ± 1.305			3.23 ± 1.261			3.40 ± 1.300			3.63 ± 1.165		
	University				3.29 ± 1.296			3.13 ± 1.323			3.23 ± 1.332			3.70 ± 1.146		
	Master and above				2.80 ± 1.328			2.61 ± 1.276			2.74 ± 1.472			3.39 ± 1.321		
**Occupation**	Legislators, government officials, community leaders,	4.43 ± 0.573	2.556 **		3.36 ± 1.367	28.618 ***		2.96 ± 1.478	35.487 ***		3.32 ± 1.492	37.538 ***		3.82 ± 1.249	3.575 ***	
	business leaders and managers															
	Professionals	4.28 ± 0.743			3.59 ± 1.233			3.38 ± 1.235			3.45 ± 1.328			3.79 ± 1.175		
	Technicians and support professionals	4.18 ± 0.804			3.58 ± 1.148			3.33 ± 1.199			3.49 ± 1.212			3.72 ± 0.982		
	Clerks	4.17 ± 0.937			3.06 ± 1.375			2.86 ± 1.374			2.99 ± 1.418			3.72 ± 1.211		
	Service and sales stuff	3.94 ± 1.030			3.16 ± 1.344			2.91 ± 1.322			3.19 ± 1.331			3.68 ± 1.204		
	Handicrafts-man	4.41 ± 0.666			3.91 ± 0.971			3.73 ± 0.985			3.86 ± 1.082			3.82 ± 1.006		
	Students	3.93 ± 0.985			2.62 ± 1.274			2.60 ± 1.290			2.69 ± 1.353			3.29 ± 1.267		
	Others	4.17 ± 0.774			2.05 ± 0.930			1.45 ± 1.113			1.55 ± 1.186			4.20 ± 1.050		
	Unemployed	4.10 ± 0.878			1.96 ± 0.984			1.53 ± 0.941			1.50 ± 0.947			3.82 ± 1.315		

* *p*<0.05, ** *p*<0.01, *** *p*<0.001, ^a^ T test/ANOVA, ^b^ Correlation.

**Table 4:** Other personal information & Risk perception.

Item	Option	Overall Impact			Property Damage			Health Effect			Life Threat			Fear Level		
		Mean ± SD	t/F ^a^	r ^b^	Mean ± SD	t/F ^a^	r ^b^	Mean ± SD	t/F ^a^	r ^b^	Mean ± SD	t/F ^a^	r ^b^	Mean ± SD	t/F ^a^	r ^b^
**Health condition**	Relatively poor	4.00 ± 1.044	5.035 **	0.122 **	3.17 ± 1.193	2.941 *	0.088 **	3.08 ± 1.165	4.790 **	0.066 **	3.17 ± 1.267	3.429 **	0.051		1.763	0.01
	Fair	3.99 ± 0.956			2.58 ± 1.246			2.21 ± 1.293			2.36 ± 1.454					
	Relatively good	4.04 ± 0.858			2.88 ± 1.281			2.75 ± 1.336			2.84 ± 1.395					
	Very good	4.26 ± 0.831			2.99 ± 1.405			2.66 ± 1.509			2.74 ± 1.550					
**Place of residence**	Macao		0.327		2.80 ± 1.324	4.455 **		2.51 ± 1.422	6.399 ***		2.61 ± 1.499	6.685 ***			0.829	
	Zhuhai				3.27 ± 1.248			3.10 ± 1.269			3.00 ± 1.311					
	Guangdong				3.22 ± 1.402			3.07 ± 1.421			3.40 ± 1.377					
	Others				2.88 ± 1.654			2.82 ± 1.380			2.71 ± 1.448					
**Whether to live in areas susceptible to typhoon**			−2.592 *			−3.580 ***			−4.527 ***			−4.161 ***			−0.611	
**Floor**	Ground level and below		0.841	−0.042	3.92 ± 1.382	5.758 ***	0.067 *	3.69 ± 1.653	7.699 ***	0.077 *	3.54 ± 1.664	6.416 ***	0.068 *	4.31 ± 1.182	3.254 **	−0.121 **
	First floor				2.41 ± 1.208			1.91 ± 1.387			1.98 ± 1.476			4.12 ± 1.150		
	2nd–7th floor				2.82 ± 1.334			2.61 ± 1.436			2.74 ± 1.501			3.73 ± 1.223		
	8th–13th floor				3.03 ± 1.361			2.72 ± 1.389			2.80 ± 1.463			3.70 ± 1.196		
	14th–19th floor				3.36 ± 1.195			3.19 ± 1.163			3.23 ± 1.213			3.63 ± 0.972		
	20th–26th floor				2.85 ± 1.313			2.58 ± 1.293			2.63 ± 1.327			3.45 ± 1.241		
	27th floor and above				2.77 ± 1.454			2.63 ± 1.424			2.61 ± 1.542			3.35 ± 1.561		
**Time to stay**	From birth		2.178	0.007	2.61 ± 1.415	12.020 ***	−0.095 **	2.44 ± 1.418	20.923 ***	−0.166 **	2.79 ± 1.440	16.078 ***	−0.172 **	3.41 ± 1.303	9.465 ***	0.150 **
	Over 10 years				3.14 ± 1.288			3.00 ± 1.303			2.99 ± 1.355			3.56 ± 1.169		
	5–10 years				3.49 ± 1.151			3.31 ± 1.223			3.39 ± 1.226			3.75 ± 1.080		
	1–5 years				2.86 ± 1.389			2.73 ± 1.336			2.79 ± 1.443			3.55 ± 1.259		
	Less than 1 year				2.64 ± 1.284			2.17 ± 1.461			2.28 ± 1.549			4.04 ± 1.155		
**Number of family members under 14**	0		0.426	0.019	2.38 ± 1.220	42.244 ***	0.332 **	2.05 ± 1.303	49.192 ***	0.355 **	2.14 ± 1.399	44.525 ***	0.336 **		1.097	0.039
	1				3.30 ± 1.267			3.12 ± 1.290			3.27 ± 1.389					
	2				3.53 ± 1.286			3.29 ± 1.325			3.28 ± 1.251					
	3				3.68 ± 1.030			3.52 ± 1.262			3.52 ± 1.327					
	4 and above				3.30 ± 1.218			3.25 ± 1.293			3.60 ± 1.231					
**Number of family members above 65**	0	4.05 ± 0.907	4.113 **	0.114 **	2.47 ± 1.216	43.191 ***	0.383 **	2.19 ± 1.332	39.716 ***	0.368 **	2.25 ± 1.404	42.220 ***	0.362 **		1.68	0.079 *
	1	4.20 ± 0.816			3.15 ± 1.344			2.96 ± 1.414			3.12 ± 1.415					
	2	4.32 ± 0.770			3.67 ± 1.197			3.39 ± 1.230			3.60 ± 1.267					
	3	4.05 ± 1.026			3.89 ± 1.243			3.58 ± 1.121			3.32 ± 1.204					
**Family members with limited mobility**			2.357 *			9.496 ***			10.333 ***			10.130 ***			2.246 *	
**Personal monthly income**	≤4999		1.676	0.031	2.24 ± 1.076	27.965 ***	0.247 *	1.88 ± 1.199	32.895 ***	0.251 **	1.91 ± 1.239	35.696 ***	0.275 **		1.313	−0.035
	5000–9999				3.24 ± 1.366			3.08 ± 1.367			3.26 ± 1.385					
	10,000–14,999				3.67 ± 1.131			3.55 ± 1.203			3.57 ± 1.303					
	15,000–19,999				3.43 ± 1.279			3.10 ± 1.274			3.35 ± 1.281					
	20,000–24,999				3.45 ± 1.276			3.18 ± 1.287			3.31 ± 1.350					
	25,000–29,999				3.07 ± 1.163			3.12 ± 1.219			3.44 ± 1.181					
	30,000–39,999				2.76 ± 1.422			2.63 ± 1.460			2.53 ± 1.466					
	40,000–59,999				3.14 ± 1.456			2.76 ± 1.480			3.03 ± 1.500					
	≥60,000				3.52 ± 1.418			3.04 ± 1.338			3.28 ± 1.487					

* *p* < 0.05, ** *p* < 0.01, *** *p* < 0.001, ^a^ T test/ANOVA, ^b^ Correlation.

**Table 5:** Knowledge, Information channels, risk perception & Active response to typhoon.

		Overall Impact		Property Damage		Health Effects		Life Threat		Fear Level	
		F ^a^	r ^b^	F ^a^	r ^b^	F ^a^	r ^b^	F ^a^	r ^b^	F ^a^	r ^b^
**Knowledge**	**Knowledge of typhoon signal**	3.983 *	−0.035	2.649	0.072 *	1.899	0.043	2.813	0.02	9.351 ***	−0.079 *
	**Understanding of typhoon prevention**	2.622 *	−0.077 *	14.332 ***	−0.182 ***	9.886 ***	−0.138 ***	8.068 ***	−0.120 ***	3.692 **	−0.063 *
	**Knowledge of typhoon preparedness**	2.592 **	0.061	9.098 ***	−0.187 ***	14.833 ***	−0.202 ***	13.129 ***	−0.213 ***	3.286 **	0.054
	**Knowledge (Weighted score)**		−0.037		−0.142 **		−0.146 **		−0.158 **		−0.065 *
**Numbers of information channels**		3.396 **	0.124 ***	6.485 ***	−0.04	13.455 ***	−0.112 ***	15.175 ***	−0.139 ***	10.599 ***	0.246 ***
**Active response to typhoon**		13.353 ***	0.202 **	21.593 ***	0.277 **	23.674 ***	0.259 **	21.012 ***	0.247 **	12.529 ***	0.204 **

* *p* < 0.05, ** *p* < 0.01, *** *p* < 0.001, ^a^ ANOVA, ^b^ Correlation.
